# Differential effects of interleukin-17 receptor signaling on innate and adaptive immunity during central nervous system bacterial infection

**DOI:** 10.1186/1742-2094-9-128

**Published:** 2012-06-15

**Authors:** Debbie Vidlak, Tammy Kielian

**Affiliations:** 1 Department of Pathology and Microbiology, University of Nebraska Medical Center, 985900 Nebraska Medical Center, Omaha, NE, 68198, USA; 2 Department of Pathology and Microbiology, University of Nebraska Medical Center, 985900 Nebraska Medical Center, Omaha, NE, 68198-5900, USA

**Keywords:** Brain abscess, IL-17R, Macrophages, γδ T cells, Neutrophils, NKT cells

## Abstract

Although IL-17A (commonly referred to as IL-17) has been implicated in the pathogenesis of central nervous system (CNS) autoimmune disease, its role during CNS bacterial infections remains unclear. To evaluate the broader impact of IL-17 family members in the context of CNS infection, we utilized IL-17 receptor (IL-17R) knockout (KO) mice that lack the ability to respond to IL-17, IL-17F and IL-17E (IL-25). In this article, we demonstrate that IL-17R signaling regulates bacterial clearance as well as natural killer T (NKT) cell and gamma-delta (γδ) T cell infiltrates during *Staphylococcus aureus*-induced brain abscess formation. Specifically, when compared with wild-type (WT) animals, IL-17R KO mice exhibited elevated bacterial burdens at days 7 and 14 following *S. aureus* infection. Additionally, IL-17R KO animals displayed elevated neutrophil chemokine production, revealing the ability to compensate for the lack of IL-17R activity. Despite these differences, innate immune cell recruitment into brain abscesses was similar in IL-17R KO and WT mice, whereas IL-17R signaling exerted a greater influence on adaptive immune cell recruitment. In particular, γδ T cell influx was increased in IL-17R KO mice at day 7 post-infection. In addition, NK1.1^high^ infiltrates were absent in brain abscesses of IL-17R KO animals and, surprisingly, were rarely detected in the livers of uninfected IL-17R KO mice. Although IL-17 is a key regulator of neutrophils in other infection models, our data implicate an important role for IL-17R signaling in regulating adaptive immunity during CNS bacterial infection.

## Introduction

Brain abscesses typically develop following parenchymal colonization with pyogenic bacteria, such as *Staphylococcus aureus* or *streptococcus* strains [1,2]. Characterized by an acute edematous response, *S. aureus* abscesses begin as localized areas of inflammation, evolving into suppurative lesions surrounded by a fibrotic capsule. Despite recent therapeutic advances, brain abscesses are still associated with significant morbidity and mortality [3]. In addition, long-term morbidity issues arise in patients recovering from these infections as a result of the extensive parenchymal damage typically associated with brain abscess formation, which can manifest as seizures, cognitive deficits, and/or hemiparesis [3-5]. Because of the ubiquitous nature of bacteria and the continuous emergence of multi-drug resistant isolates, such as methicillin-resistant *S. aureus* (MRSA), these central nervous system (CNS) infections are likely to persist [6-8]. Therefore, a better understanding of the complex host-pathogen interactions that occur during brain abscess formation is essential for the development of novel therapies to treat these devastating infections.

The role of T helper 17 (Th17) cells in various inflammatory diseases has been a topic of intense investigation in recent years. Although Th17 cells have been implicated in the pathogenesis of autoimmune diseases [9-13], they have also been shown to provide protection against extracellular bacterial infections [14-16]. IL-17A (commonly referred to as IL-17) is the prototypic cytokine of the IL-17 family which includes six members, namely IL-17A, B, C, D, E and F [17-21]. In general, IL-17 plays an important role in regulating tissue inflammation through the induction of proinflammatory cytokines (IL-6, TNF-α and IL-1β) [22,23], chemokines (CXCL1, CXCL6, CXCL8 and CCL2) [24], and metalloproteinases [25] from various cell types, resulting in neutrophil recruitment [9,19,26]. To date, five IL-17 receptors (IL-17Rs) have been described (IL-17RA, -B, -C, -D and -E) [22,27-29], with mRNA ubiquitously expressed in a wide array of tissues (for example, lungs, kidney, liver, spleen and brain) [22,27] and multiple cell types (for example, macrophages, lymphocytes, fibroblasts and epithelial cells) [27]. The binding of IL-17 family members to their corresponding receptors triggers a signaling cascade that elicits cytokine and chemokine production. Because IL-17, IL-17E, and IL-17F all signal via the IL-17R [9,30,31], analysis of IL-17R knockout (KO) mice represents a way of investigating the broader impact of IL-17 family member action within the CNS. This was the rationale employed in the current study.

Among numerous proinflammatory mediators, IL-17 expression is elevated during brain abscess development [32]. Recent work from our laboratory has demonstrated that Th17 cells are critical for bacterial containment and regulation of innate immune cell infiltrates during the later stages of brain abscess formation [33,34]. This finding suggests that IL-17 may be a key determinant in regulating immune responses during CNS bacterial infection, but this remains to be determined as Th17 cells secrete other inflammatory cytokines that can affect the course of inflammation (for example, IL-22 and IL-27). Therefore, the current study was designed to evaluate the functional role of IL-17 family members in brain abscess pathogenesis by utilizing IL-17R KO mice. Our data revealed that IL-17R KO mice displayed higher bacterial burdens than wild-type (WT) animals, but that this had no effect on survival following CNS *S. aureus* infection. In accordance with the elevated expression of select inflammatory mediators (for example, CXCL2 and CXCL9), we also detected increased gamma-delta (γδ) T cell infiltrates in the brains of IL-17R KO mice, suggesting a potential compensatory mechanism in the absence of IL-17R signaling. Most notably, these studies describe an apparent natural killer T (NKT) cell deficiency in IL-17R KO animals, a novel finding that may offer insights into *S. aureus* CNS infection, as well as other peripheral models of infection and injury.

## Materials and methods

### Mice

IL-17RA KO mice (C57BL/6 background) were obtained from Amgen (Seattle, WA, USA) [9]. Age- and sex-matched C57BL/6 mice (Charles River Laboratories, Frederick, MD, USA, through a contract with National Cancer Institute) were used as WT controls. All animals were bred and housed in an AAALAC-accredited animal facility at the University of Nebraska Medical Center, provided with food and water *ad libitum*, and housed under 12 h light/dark cycles. Brain abscess studies were performed with mice between 10 and 16 weeks of age.

### Generation of experimental brain abscesses

Brain abscesses were induced by intracerebral injection of a MRSA USA300 strain encapsulated in agarose beads as previously described [35]. This isolate was recovered from an otherwise healthy individual who died from a brain abscess [36]. It is important to note that MRSA strains are uncommonly observed in community-acquired brain abscesses [36], whereas they are more prevalent in infections arising after trauma or neurosurgical procedures [37], and may differ in virulence compared to methicillin-sensitive *S. aureus* (MSSA). Briefly, mice were anesthetized with an intraperitoneal injection of 2.5% avertin. A 1 cm longitudinal incision was then made in the scalp to expose the underlying skull sutures and facilitate the identification of bregma. A rodent stereotaxic apparatus equipped with a Cunningham mouse adaptor (Stoelting, Kiel, WI, USA) was used to implant *S. aureus*-encapsulated beads into the striatum, using the following coordinates relative to bregma: +1.0 mm rostral, +2.0 mm lateral, and -3.0 mm deep from the surface of the brain. A burr hole was made and a 10 μl Hamilton syringe fitted with a 26-gauge needle was used to slowly deliver 2 μl of *S. aureus*-laden beads (7 × 10^3^-1 × 10^4^ colony forming units (CFU)) into the brain parenchyma. The needle remained in place for 2.5 minutes following injection to minimize bead efflux and potential leakage into the meninges. The skin incision was closed using surgical glue and animals were closely monitored over the course of each study for clinical indices of infection. The animal-use protocol, approved by the University of Nebraska Medical Center Animal Care and Use Committee, is in accord with the National Institutes of Health guidelines for the use of rodents.

### Quantitation of viable bacteria from brain abscesses

To quantitate the numbers of viable *S. aureus* associated with brain abscesses *in vivo*, serial ten-fold dilutions of brain abscess homogenates were plated onto modified trypticase soy agar plates (Becton Dickinson, Sparks, MD, USA) supplemented with 5% defibrinated sheep blood (Hemostat Laboratories, Dixon, CA, USA). Titers were calculated by enumerating colonies and are expressed as CFU per gram of tissue.

### Histological analysis of brain tissues

Immediately *ex vivo*, brains from IL-17R KO and WT mice were placed in a cryomold (Fisher Scientific, Fair Lawn, NJ, USA), embedded in Optimal Cutting Temperature (OCT) medium (Tissue-Tek, Torrance, CA, USA), and placed on dry ice until frozen. Cryostat sections (15 μm) were mounted onto glass slides (Erie Scientific Co., Portsmouth, NH, USA) and subjected to H&E staining (Fisher Scientific, Fair Lawn, NJ, USA). Images (20×) were collected using a digital slide scanner (Ventana Medical Systems, Tucson, AZ, USA) and the final images (1×) were prepared using Ventana Medical Image View Software.

### Multi-analyte microbead array for detection of proinflammatory mediator production

To quantitate inflammatory mediator production in brain abscess homogenates, a mouse 19-plex microbead suspension array system was used according to the manufacturer’s instructions (Millipore Corporation, Billerica, MA, USA). This customized array allows for the simultaneous detection of 19 individual inflammatory molecules in a single 50 μl sample, including IL-1α, IL-1β, TNF-α, IFN-γ, IL-6, IL-9, IL-10, IL-12 p40 and p70, IL-15, IL-17, CCL2, CCL3, CCL4, CCL5, CXCL1, CXCL2, CXCL9 and CXCL10. Results were analyzed using a Bio-Plex Workstation (Bio-Rad, Hercules, CA, USA) and adjusted based on the amount of total protein extracted from abscess tissues for normalization. The level of sensitivity for most analytes in the array was 3.2 pg/ml.

### Quantitation of abscess-associated cells by fluorescence-activated cell sorting

To determine whether IL-17R signaling affected innate and/or adaptive immune cell influx into brain abscesses, cell populations were quantitated by fluorescence-activated cell sorting (FACS) as previously described [35,38,39]. Briefly, mice were manually perfused with isotonic PBS, pH 7.4, for approximately 2 minutes (at approximately 30 ml/minute) to eliminate leukocytes from the vasculature until the liver appeared blanched. Prior histological analysis had demonstrated the absence of leukocytes remaining adherent to the cerebral vascular endothelium and lack of perivascular cuffing in the Virchow Robin space of vascular-perfused mice in the brain abscess model (data not shown). Based on these observations, FACS analysis is an accurate representation of cells that have invaded the CNS parenchyma. Following vascular perfusion, the entire infected hemisphere was collected to recover abscess-associated cells, which ensured that equivalent tissue regions were obtained from both IL-17R KO and WT mice for downstream comparisons of leukocyte infiltrates. Tissues were minced in Hank’s Balanced Salt Solution (HBSS; Hyclone Laboratories, Logan, UT, USA) supplemented with 10% Fetal Bovine Serum (FBS; Atlanta Biologicals, Lawrenceville, GA, USA) and filtered through a 70 μm nylon mesh cell strainer. At this point, an aliquot of tissue homogenate from each animal was collected to quantitate bacterial burdens. The resulting slurry was then digested for 30 minutes at 37 °C in HBSS supplemented with 0.2 mg/ml collagenase type I and 28 U/ml DNase I (both from Sigma-Aldrich, St Louis, MO, USA) to obtain a single-cell suspension. Following enzyme neutralization, cells were layered onto a discontinuous Percoll gradient (1.03-1.088 g/ml) and centrifuged at 2,400 rpm for 20 minutes at room-temperature in a swinging bucket rotor. After centrifugation, myelin debris was carefully aspirated and the cell interface collected. Following extensive washes and incubation in Fc Block™ (BD Biosciences, San Diego, CA, USA), a panel of directly-conjugated antibodies was used for multi-color FACS to identify neutrophils (F4/80^-^, CD45^+^, Ly6G^+^), macrophages (F4/80^+^, CD45^high^, Ly6G^-^), microglia (F4/80^+^, CD45^low-intermediate^, Ly6G^-^), CD4 T cells (CD3^+^CD4^+^), CD8 T cells (CD8a^+^), NK cells (NKp46^+^, NK1.1^+^), NKT cells (CD3^+^, NKp46^-^, NK1.1^+^), and γδ T cells (γδTCR^+^). A recent study from our laboratory has established that the majority of CD4^+^ infiltrates during brain abscess development are Th1 and Th17 cells as shown by CD3^+^ co-expression [34]. Antibodies were purchased from the following vendors: F4/80-AlexaFluor488 (AbD Serotec, Raleigh, NC, USA); CD45-APC, Ly-6G-PE, CD4-AlexaFluor700, CD8a-FITC, and NK1.1-APC (BD Biosciences); and NKp46-PE and γδTCR-PE-Cy5 (eBioscience, San Diego, CA, USA). Cells were analyzed using a BD LSRII (BD Biosciences) with compensation based on the staining of each individual fluorochrome alone and correction for autofluorescence with unstained cells. Controls included cells stained with isotype control antibodies to assess the degree of non-specific staining. Analysis was performed using BD FACSDiva™ software (BD Biosciences) with cells gated on the total leukocyte population. Results are presented as the absolute number of cells recovered from each brain, with normalization to adjust for the recovery of different cell numbers from the two mouse strains.

### Statistics

Significant differences in bacterial titers, immune cell infiltrates, and proinflammatory mediator expression between IL-17R KO and WT mice at a particular time point were determined using a paired Student’s *t*-test (SPSS Science, Chicago, IL, USA). A *P*-value of less than 0.05 was considered statistically significant.

## Results

### IL-17 receptor signaling is important for bacterial clearance during central nervous system *S. aureus* infection

**Figure 1:**
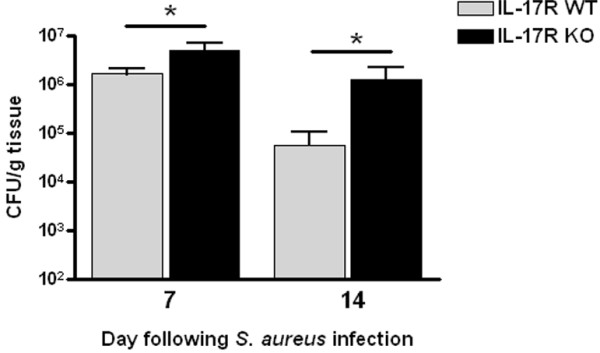
**IL-17 plays an important role in bacterial clearance during*S. aureus *brain abscess formation.** Brain abscess tissues from IL-17 receptor (IL-17R) knockout (KO) and wild-type (WT) mice (n = 5 to 8 per group) were collected at days 7 and 14 after infection. Bacterial burdens were then determined by quantitative culture. Results are expressed as colony forming units (CFU) of *S. aureus* per gram of wet tissue weight (mean ± SEM) combined from four independent experiments. *P < 0.05, significant differences in bacterial titers between IL-17R KO and WT mice.

**Figure 2:**
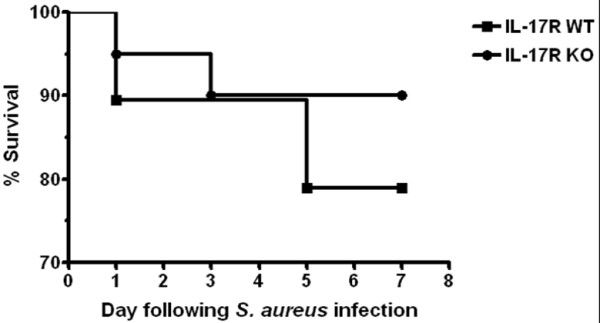
**IL-17 receptor signaling does not affect survival during central nervous system bacterial infection.** Brain abscesses were induced in IL-17 receptor (IL-17R) knockout (KO) and wild-type (WT) mice (n = 5 to 8 per group) and animals were monitored daily after infection. Any moribund mice were euthanized and dates recorded. Results are presented as the percent (%) survival and are representative of three independent experiments.

**Figure 3:**
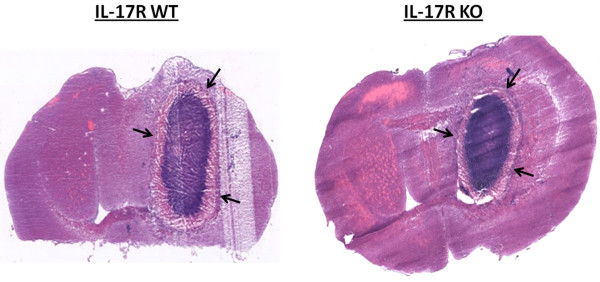
**Loss of IL-17 receptor signaling does not affect the extent of tissue damage during brain abscess development.** Brain tissues from IL-17 receptor (IL-17R) knockout (KO) and wild-type (WT) mice (n = 6 to 8 per group) were collected at day 7 following *S. aureus* infection, whereupon tissues were sectioned for H&E staining to evaluate abscess sizes (arrows). Results are representative of two independent experiments.

Previous work from our laboratory has established significant IL-17 production and Th17 infiltrates during brain abscess development [32,34]. Based on these observations, it was expected that IL-17 family members would be an essential component in the developing immune response to *S. aureus* brain abscesses, since IL-17A and IL-17E are known to play a role in granulopoiesis, neutrophil recruitment, and proinflammatory cytokine production in response to extracellular bacterial infections [40,41]. To investigate the functional importance of IL-17 signaling in the context of CNS infection, brain abscess pathogenesis was evaluated in IL-17R KO and WT mice. IL-17R signaling was found to be important in controlling bacterial clearance, as brain abscess tissues of IL-17R KO animals displayed significantly higher bacterial burdens at days 7 and 14 after infection compared with WT mice (Figure 1). Despite greater numbers of bacteria present in the brains of IL-17R KO mice, IL-17R signaling had no significant effect on survival in this model (Figure 2). Histological analysis of brain abscesses from IL-17R KO and WT animals did not reveal any significant alterations in lesion size between the two groups (Figure 3). In addition, no differences in edema formation were observed between IL-17R KO and WT mice at both the gross or histological levels, and abscess wet tissue weights between both groups were nearly identical (data not shown). Collectively, these findings suggest that edematous responses are not exaggerated in IL-17R KO animals following CNS bacterial infection.

### Loss of IL-17 receptor signaling results in elevated proinflammatory mediator production in brain abscesses

**Figure 4:**
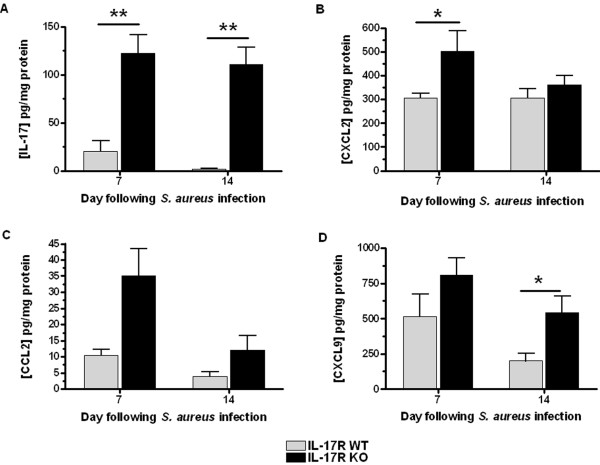
**Loss of IL-17 receptor signaling results in elevated proinflammatory mediator production.** Supernatants were collected from brain abscess homogenates of IL-17 receptor (IL-17R) knockout (KO) and wild-type (WT) mice (n = 5 to 8 per group) at days 7 and 14 after infection, whereupon IL-17 **(a)**, CXCL2 **(b)**, CCL2 **(c)** and CXCL9 **(d)** production was quantitated using multi-analyte bead arrays. Results are representative of two independent experiments and are presented as the average cytokine/chemokine concentration normalized to the total amount of protein (mean ± SD). *P < 0.05, **P < 0.01, significant differences between IL-17R KO and WT mice.

The fact that IL-17 is known to induce neutrophil chemokine expression, in conjunction with our previous findings that IL-17-producing CD4^+^ T cells are the predominant T cell subset associated with brain abscesses [42], led us to predict that proinflammatory mediator production would be attenuated with the loss of IL-17R signaling. Surprisingly, the expression of several proinflammatory mediators was enhanced in IL-17R KO mice. While IL-17R KO animals predictably had significantly higher amounts of IL-17 in brain abscess homogenates (Figure 4A), several other mediators were also elevated between 7 and 14 days after infection, including IL-1α, IL-1β, IL-6, CCL3 and CXCL1 (data not shown), as well as CXCL2 and CXCL9 (Figure 4B,D). These findings suggest that IL-17R signaling influences inflammatory mediator production on a more global scale, which may result from the inability of IL-17R KO mice to efficiently clear *S. aureus* from the CNS.

### Innate immune cell recruitment into brain abscesses proceeds in an IL-17-independent manner

**Figure 5:**
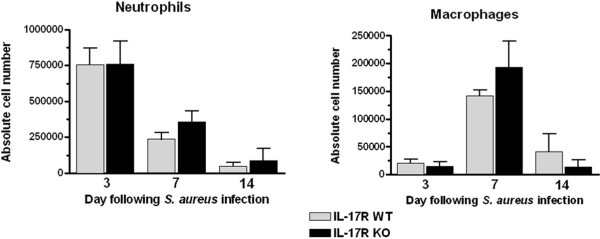
**IL-17 receptor signaling does not influence neutrophil or macrophage recruitment during central nervous system*S. aureus *infection.** Abscess-associated cells from IL-17 receptor (IL-17R) knockout (KO) and wild-type (WT) mice (n = 5 to 9 per group) were recovered at the indicated time points after infection and analyzed by flow cytometry to identify neutrophils and macrophages. Results are presented as the absolute cell number of each population recovered from a total of three independent experiments (mean ± SD).

A primary role of IL-17 during tissue inflammation is neutrophil recruitment, mediated indirectly by the ability of IL-17 to induce neutrophil chemokine release [9,19,26]. However, the effect of IL-17R signaling on immune cell influx into the infected CNS is not known. To investigate this relationship, FACS analysis was performed on brain abscess tissues from IL-17R KO and WT mice. Despite the important role that IL-17 plays in neutrophil recruitment in other model systems, it was not required during CNS *S. aureus* infection since IL-17R KO mice were as equally capable as WT animals of recruiting these cells to the site of infection (Figure 5A). The same trend was observed with macrophage infiltrates (Figure 5B), and no noticeable differences in the absolute numbers of microglia were observed in these studies (data not shown). Collectively, these results indicate that IL-17R signaling has minimal effects on innate immune infiltrates during the course of CNS *S. aureus* infection.

### IL-17 receptor loss differentially regulates T cell subset infiltration into the infected central nervous system

**Figure 6:**
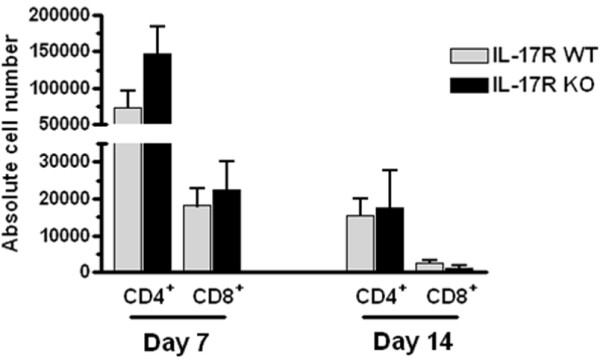
**IL-17 receptor loss has no effect on T cell recruitment into brain abscesses.** Abscess-associated cells from IL-17 receptor (IL-17R) knockout (KO) and wild-type (WT) mice (n = 5 to 9 per group) were recovered at the indicated time points after infection, and analyzed by flow cytometry to identify CD4^+^ and CD8^+^ T cells. Results are presented as the absolute cell number for each population recovered from a total of four independent experiments (mean ± SD).

**Figure 7:**
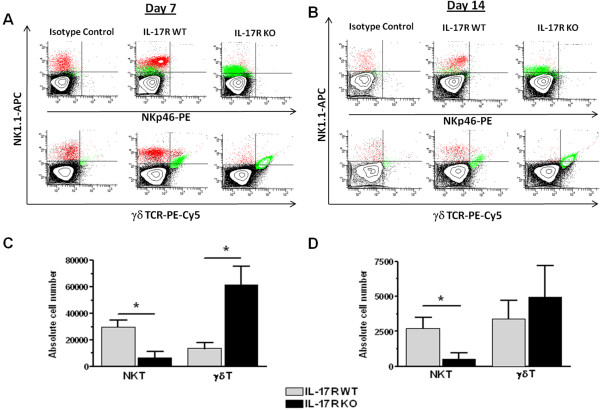
**IL-17 receptor signaling differentially regulates natural killer T (NKT) and gamma-delta (γδ) T cell recruitment into brain abscesses.** Abscess-associated cells from IL-17 receptor (IL-17R) knockout (KO) and wild-type (WT) mice (n = 5 to 9 per group) were recovered at the indicated time points after infection and analyzed by flow cytometry to identify NKT (NK1.1^+^/NKp46^-^; red) and gamma-delta (γδ) T cells (γδTCR^+^; green). Fluorescence-activated cell sorting (FACS) contour plots are presented to demonstrate the different population densities and distributions between IL-17R KO and WT mice at days 7 **(a)** and 14 **(b)**. These populations were quantified and results presented as the absolute cell number of each population at days 7 **(c)** and 14 **(d)** following infection from a total of four independent experiments (mean ± SD). *P < 0.05, significant differences in abscess-associated T cell subsets between IL-17R KO and WT mice.

While previous studies from our laboratory have established the presence of IL-17-producing T cells during brain abscess development [32,34], we have yet to define how the action of IL-17R influences the accumulation of various T cell subsets. To address this issue, FACS analysis was performed on brain abscess tissues from IL-17R KO and WT animals. As previously demonstrated, CD4^+^ T cells were the most frequent T cell infiltrate, with fewer CD8^+^ T cells (Figure 6); however, no significant differences in the absolute numbers of CD4^+^ or CD8^+^ T cells were observed between IL-17R KO and WT mice (Figure 6). We expanded our analysis to include less frequent brain abscess T cell infiltrates, including NKT cells and γδ T cells (Figure 7). Interestingly, NKT cells were significantly reduced in brain abscesses of IL-17R KO mice throughout infection (Figure 7A,B), whereas the opposite was true of γδ T cells, which were significantly elevated compared with WT animals at day 7 post-infection (Figure 7A). In contrast, no significant differences in NK cell infiltrates were detected between groups (data not shown). These findings suggest that the loss of IL-17R signaling has the greatest effect on T cell populations that span innate-adaptive immunity (that is, NKT cells and γδ T cells) in the context of CNS *S. aureus* infection.

### IL-17 receptor knockout mice exhibit defects in natural killer T cell populations

**Figure 8:**
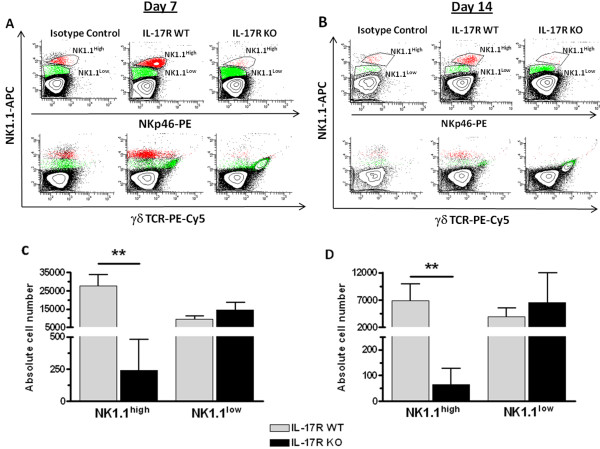
**Differential expression of NK1.1 in abscess-associated tissue.** Abscess-associated cells from IL-17 receptor (IL-17R) knockout (KO) and wild-type (WT) mice (n = 5 to 9 per group) were recovered at days 7 **(a)** and 14 **(b)** after infection and analyzed by flow cytometry to identify NK1.1^high^ and NK1.1^low^ infiltrates. Representative contour plots depict differences in population densities between IL-17R WT and KO mice. Post-analysis gates were drawn around NK1.1^high^ (red) and NK1.1^low^ (green) populations in the top panel (NK1.1 versus NKp46) and corresponding populations can be seen in the bottom panel (NK1.1 versus γδ TCR). These populations were quantified at days 7 **(c)** and 14 **(d)** after infection and are expressed as the absolute number of each population recovered from four independent experiments (mean ± SD). **P < 0.01, significant differences between IL-17R KO and WT mice.

**Figure 9:**
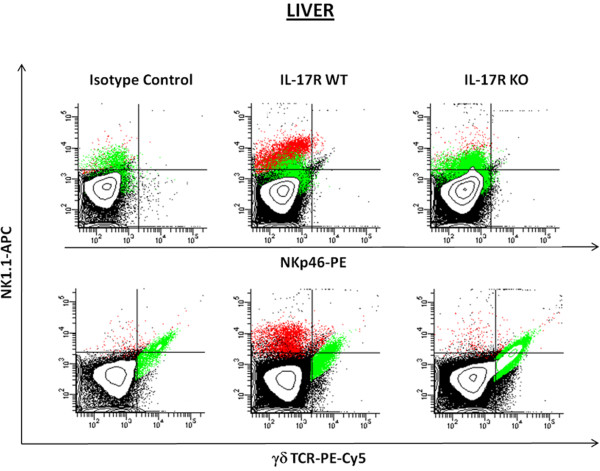
**NK1.1**^**high **^**cells are rarely detected in the livers of IL-17 receptor knockout mice.** Cells were collected from the livers of uninfected IL-17 receptor (IL-17R) knockout (KO) and wild-type (WT) mice (n = 3 to 6 per group) and analyzed by flow cytometry to identify NKT and gamma-delta (γδ) T cells. Fluorescence-activated cell sorting (FACS) contour plots are presented to demonstrate the different population densities and distributions between IL-17R WT and IL-17R KO mice. The total NK1.1^+^ population is indicated in red, whereas the total γδ TCR^+^ population is indicated in green. Plots are representative of three independent experiments.

Closer examination of our flow cytometry data revealed the presence of two distinct NK1.1^+^ populations in the brain, namely NK1.1^high^ and NK1.1^low^ (Figure 8A,B). While there was little difference in NK1.1^low^ infiltrates between groups, the NK1.1^high^ population was significantly reduced in IL-17R KO mice, to the point of being difficult to detect in some experiments (Figure 8C,D). Based on these findings, we examined whether the failure to detect NK1.1^high^ infiltrates in brain abscesses resulted from a defect in recruitment or an inherent absence of these cells in the periphery. Therefore, the frequency of NK1.1^high^ cells was examined in the livers of uninfected IL-17R KO and WT mice as the majority of NK1.1^high^ cells reside in the liver [43]. Interestingly, NK1.1^high^ cells were rarely detected in the livers of non-manipulated IL-17R KO mice (Figure 9). To our knowledge, the requirement for IL-17R signaling in populating the liver with NK1.1^high^ cells has not yet been reported in the literature and could offer important insights into the relationship between IL-17R and T cell function in various animal models. However, since recent studies from our laboratory have demonstrated that NKT cells do not play a significant role in regulating immune responses during brain abscess development (Holley and Kielian, unpublished observations), this avenue was not pursued further.

## Discussion

The role of IL-17 and its receptor have been well studied in recent years [20,44-46]. Numerous cell types are known to produce IL-17, including γδ, NKT and CD4^+^ T cells [47-49], resulting in robust inflammatory mediator production and subsequent neutrophil accumulation [22-24,49,50]. While IL-17 production is often associated with various autoimmune disorders [9-13], the cytokine is also known to exert protective effects during extracellular bacterial infections [14-16]. Our previous studies have demonstrated significant IL-17 production during brain abscess development [32-34], and Th17 cells represent a predominant CD4^+^ infiltrate. To understand the functional importance of IL-17 and related family members on brain abscess pathogenesis, we examined disease progression in IL-17R KO mice. Given its key role in neutrophil recruitment, and our previous findings that neutrophils are essential for survival during *S. aureus* abscess formation [51], we expected that loss of IL-17R signaling would result in a diminished capacity to control infection. To some extent this prediction was correct, as shown by the fact that IL-17R KO mice exhibited delayed bacterial clearance compared with WT animals. However, despite elevated bacterial burdens, survival rates were similar between IL-17R KO and WT animals. An explanation for the latter observation is that, despite the defect in IL-17R function, IL-17R KO mice were as effective as WT animals in recruiting innate immune cells into the infected brain. As established by previous studies in our laboratory [51], a correlation exists between the degree of neutrophil infiltration and bacterial titers in the brain. Therefore, despite the elevated bacterial burdens observed in IL-17R KO mice, these animals were no more likely to succumb to infection than WT mice since they retained the ability to effectively recruit neutrophils into the brain. This suggests that an alternative signal (or signals) is capable of eliciting neutrophil chemokine expression and recruitment in the absence of IL-17R action. Indeed, many redundant mechanisms exist to ensure efficient pathogen recognition and clearance by the host immune system, with possible candidates including complement split products (for example, C3a, C5a) and bacterial components (for example, formylated peptides). Therefore, while IL-17R signaling is not essential for survival following CNS *S. aureus* challenge, it does appear to be important in controlling bacterial burdens, though the exact mechanism(s) of action remains to be determined.

Differentiation of naïve CD4^+^ T cells into T helper or effector cells relies heavily on the inflammatory milieu. As we have previously demonstrated, Th17 infiltrates are present in *S. aureus*-induced brain abscesses [33,34,42]. Based on the understanding that IL-17 plays a pivotal role in inducing and maintaining an effective immune response, as well as results from others utilizing IL-17R KO mice [9,52], we expected that a loss in IL-17R function would result in decreased proinflammatory mediator expression during CNS *S. aureus* infection. On the contrary, most mediators that we measured were elevated in brain abscesses of IL-17R KO animals. In particular, potent neutrophil chemoattractants, such as CXCL1 and CXCL2, were increased in IL-17R KO mice, possibly acting to compensate for the loss of IL-17R signaling. Evidence to support this possibility is provided by the fact that neutrophil infiltrates were similar in both IL-17R KO and WT animals. It is important to note that IL-17R KO mice have been shown to exhibit fewer circulating neutrophils in other studies [9,52]. This discrepancy may be explained by the fact that most reports using IL-17R KO mice have focused on peripheral sites of inflammation in the spleen, liver and lungs, whereas we examined a unique tissue compartment in the CNS. Since IL-17RA is responsible for transducing signals emanating from IL-17, IL-17 F and IL-17E (IL-25) [20,29], we are not able to definitively assign the biological actions of IL-17R signaling to one specific IL-17 family member. Nonetheless, the approach to begin studying IL-17R KO animals was preferred since it represented a more global means of assessing IL-17 isoform involvement, whereupon the identification of specific family members can be assessed in future studies that are outside the scope of the current report.

While the early innate response is crucial to controlling bacterial burdens and recruiting effector cells into the brain during abscess formation, equally important is the developing adaptive immune response [34]. As we and others have previously shown [33,34,53], CD4^+^ T cells are detected in brain abscesses as early at 3 days after infection and our recent study [34], using T cell adoptive transfers into TCR αβ KO mice, revealed that both Th1 and Th17 cells are important for effective bacterial clearance during brain abscess development. To address whether Th1 or Th17 infiltrates were altered in the context of IL-17R loss, we performed intracellular cytokine staining for IL-17 and IFN-γ. No significant differences in the proportions of CD3^+^CD4^+^ Th1 or Th17 cells were observed in the brains of WT and IL-17R KO mice at day 7 following *S. aureus* infection (data not shown) when peak T cell infiltrates were apparent (Figure 6). As the absolute numbers of CD4^+^ T cells in the brains of IL-17R KO and WT mice were similar at day 7 post-infection, the increased IL-17 levels observed in KO mice are likely to be attributed to either cytokine accumulation based on receptor absence or, alternatively, the significantly larger population of γδ T cells infiltrating the CNS of IL-17R KO mice that can also produce IL-17.

Most notable was the effect of IL-17R loss on non-traditional T cell populations, specifically NKT cells and γδ T cells, which are often considered transitional cells because of their ability to bridge innate and adaptive immunity [54,55]. Our studies found that γδ T cell infiltrates were significantly increased in brain abscesses of IL-17R KO mice compared with WT animals at day 7 after infection, and remained elevated through day 14. Cell surface expression of Toll-like receptor (TLR)2 on γδ T cells [56], coupled with their ability to rapidly produce proinflammatory cytokines such as IL-17 [57,58] and IFN-γ [59,60], makes γδ T cells adept as early responders to bacterial infection. However, the role that γδ T cells play during brain abscess development in the context of IL-17R loss remains uncertain.

NKT cells represent a unique lymphocyte population that expresses NK cell markers as well as a semi-invariant T cell receptor [43,61] and NKT cell infiltrates are detected during early brain abscess development [42]. Initial studies reported significantly decreased accumulation of NKT cells in brain abscesses of IL-17R KO mice compared with WT animals at both days 7 and 14 following CNS infection. Further analysis revealed the presence of two distinct NK1.1^+^ populations, namely NK1.1^high^ and NK1.1^low^. The NK1.1^low^-expressing population was found to co-express the γδ TCR, indicating that these cells were likely to be γδ T cells, albeit only a fraction of the total γδ T cell population, as the absolute numbers of NK1.1^+^, γδ TCR^+^ cells remained relatively constant throughout infection, with little difference noted between IL-17R KO and WT mice. Additionally, both the NK1.1^high^ and NK1.1^low^ cells in the brain were found to be predominantly CD4^-^ (data not shown). As these preliminary studies were designed to examine fundamental differences in infiltrating immune cells in IL-17R KO versus WT mice, a more comprehensive analysis would be required to determine whether these NK1.1^+^ populations represent classical or invariant NKT cells. However, although NKT cells represent a sizable immune cell infiltrate during early brain abscess development, recent studies from our laboratory utilizing CD1d KO mice, which lack all NKT cell subsets, failed to reveal any distinct phenotypes during CNS infection (Holley and Kielian, unpublished observations).

An intriguing finding of the current study was that NK1.1^high^ cell infiltrates were essentially absent in brain abscesses of IL-17R KO mice. We looked at whether defective NK1.1^high^ cell recruitment into brain abscesses in IL-17R KO animals resulted from impaired trafficking into the CNS or if these cells were absent in the periphery. While NK1.1^+^ cells are broadly distributed in mice, they are most frequent in the liver [43,61,62]. To determine whether IL-17R KO mice demonstrated an inherent deficiency in NK1.1^high^ cells, we analyzed liver tissues from uninfected IL-17R KO and WT animals. Interestingly, FACS analysis revealed few NK1.1^high^-expressing cells in IL-17R KO mice under resting conditions. Unlike the CD4^-^ NK1.1^high^ infiltrate in brain abscesses of WT animals, NK1.1^high^ cells in the livers of WT mice were predominantly CD4^+^ (data not shown). Several studies have described the immense diversity in NKT cell phenotypes and functionality [63-66]. The differential expression of CD4 on NK1.1^+^ cells isolated from the brain and liver of mice suggests that these may represent two functionally and potentially developmentally distinct populations. To our knowledge, this is the first report describing a paucity of NK1.1^high^ cells in IL-17R KO mice; a finding that could offer valuable insights into their function during both CNS and systemic diseases.

In summary, we have described an important role for IL-17R signaling in controlling bacterial burdens during CNS *S. aureus* infection. Despite their defect in *S. aureus* clearance, IL-17R KO mice were no more likely to succumb to infection than WT animals. This could be attributed to increased inflammatory infiltrates (that is, γδ T cells) in brain abscess of IL-17R KO mice, corresponding with an ability to control infection in an IL-17-independent manner. Finally, we describe for the first time an inherent rarity of NK1.1^high^-expressing cells in uninfected IL-17R KO mice. From a broader perspective, this finding could have important implications, as IL-17R KO mice are commonly used to study both autoimmune and infectious diseases [67,68].

## Abbreviations

CCL2, Macrophage chemoattractant protein-1/MCP-1; CCL3, Macrophage inflammatory protein-1α/MIP-1α; CXCL1, Keratinocyte chemokine/KC; CXCL2, Macrophage inflammatory protein-2/MIP-2; CXCL9, Monokine induced by IFN-γ/MIG; CFU, Colony forming units; CNS, Central nervous system; FACS, Fluorescence-activated cell sorting; γδ, Gamma-delta; H&E, Hematoxylin and eosin; IFN, Interferon; IL, Interleukin; IL-17R, IL-17 receptor; KO, Knockout; MRSA, Methicillin-resistant Staphylococcus aureus; MSSA, Methicillin-sensitive S. aureus; NK, Natural killer; NKT, Natural killer T; PBS, Phosphate-buffered saline; TH17, T helper 17; TLR, Toll-like receptor; TNF, Tumor necrosis factor; WT, Wild-type.

## Competing interests

The authors declare that they have no competing interests.

## Authors’ contributions

DV performed the experiments and data analysis, participated in study design, and helped to draft the manuscript. TK conceived the study, participated in study design and data interpretation, and helped draft and revise the manuscript. Both authors have read and approved the final version of the manuscript.
